# Hemp-Based Meat Analogs: An Updated Review on Extraction Technologies, Nutritional Excellence, Functional Innovation, and Sustainable Processing Technologies

**DOI:** 10.3390/foods14162835

**Published:** 2025-08-15

**Authors:** Hassan Barakat, Thamer Aljutaily

**Affiliations:** Department of Food Science and Human Nutrition, College of Agriculture and Food, Qassim University, Buraydah 51452, Saudi Arabia; thamer.aljutaily@qu.edu.sa

**Keywords:** hemp protein, meat analogues, functional properties, food security, agriculture sustainability

## Abstract

The global transition toward plant-based diets has intensified the search for sustainable protein alternatives, positioning hemp-based meat analogs (HBMAs) as a promising solution due to their exceptional nutritional profile and environmental benefits. This comprehensive review critically examines hemp protein research, focusing on extraction technologies, nutritional excellence, functional innovation, and sustainable processing approaches for meat analog development. Hemp seeds contain 25–30% protein, primarily consisting of highly digestible edestin and albumin proteins that provide a complete amino acid profile comparable to soy and animal proteins. The protein exhibits superior digestibility (>88%) and generates bioactive peptides with demonstrated antioxidant, antihypertensive, and anti-inflammatory properties, offering significant health benefits beyond basic nutrition. Comparative analysis reveals that while alkaline extraction-isoelectric precipitation remains the industrial standard due to cost-effectiveness ($2.50–3.20 kg^−1^), enzymatic extraction and ultrasound-assisted methods deliver superior functional properties despite higher costs. Hemp protein demonstrates moderate solubility and good emulsifying properties, though its gelation capacity requires optimization through enzymatic hydrolysis, high-pressure processing, or strategic blending with complementary proteins. Processing innovations, particularly high-moisture extrusion combined with protein blending strategies, enable fibrous structures closely mimicking conventional meat texture. Hemp protein can replace up to 60% of soy protein in high-moisture meat analogs, with formulations incorporating wheat gluten or chickpea protein showing superior textural attributes. Despite advantages in nutritional density, sustainability, and functional versatility, HBMAs face challenges including sensory limitations, regulatory barriers, and production scaling requirements. Hemp cultivation demonstrates 40–50% lower carbon footprint and water usage compared with conventional protein sources. Future research directions emphasize techniques and action processes, developing novel protein modification techniques, and addressing consumer acceptance through improved sensory properties for successful market adoption.

## 1. Introduction

The global shift towards plant-based diets has spurred innovation in meat analogs, with hemp protein emerging as a promising alternative due to its high nutritional value and sustainable cultivation [1,2]. Hemp (*Cannabis sativa* L.) is extensively utilized in industries for its durable fibers and versatile biomass, which are processed into textiles, bio-composites for automotive parts, building insulation, paper, biofuel, personal care products, and food supplements, with ongoing advancements in integrated biorefinery approaches enabling sustainable and high-quality production of fibers, oils, construction materials, and fine chemicals [3]. Interestingly, in 2024, the global hemp industry was valued at 6–11 B$, with forecasts to exceed 30 B$ by 2034, driven by legalization, sustainability, and diverse applications from textiles to CBD [4,5]. China leads production with over 73,000 t of refined fiber and top seed output, while France supplies 78% of EU fiber and expands acreage [6]. Canada, India, the Netherlands, and the US—where the hemp sector hit 445 M$ in 2024—also contribute significantly amid evolving regulations [7]. Paraguay dominates Latin America, and liberalizing markets in Africa and Asia are scaling cultivation for domestic and export demand. Future growth hinges on bio-composites, wellness products, construction materials, and other sustainable goods, supported by regulatory liberalization and processing investments [4,6].

**Figure 1:**
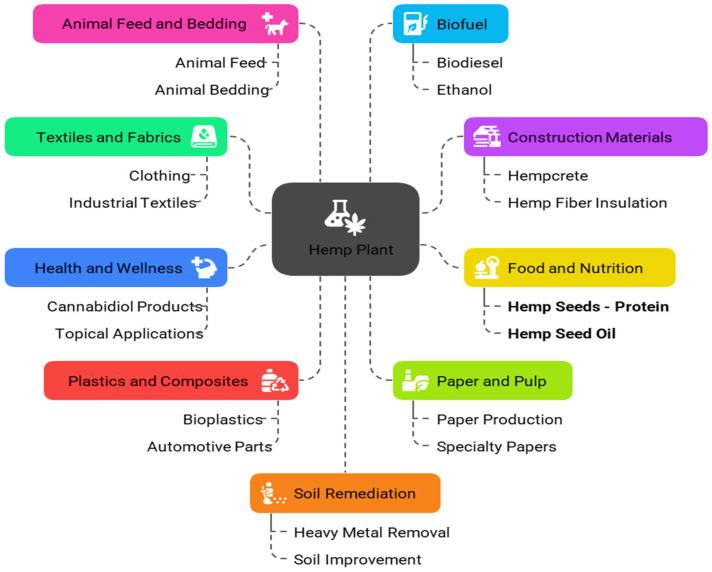
Industrial applications of hemp plants.

Industrial hemp seeds and their derivatives are increasingly incorporated into food products due to their high nutritional value, including protein, fiber, and essential fatty acids, with applications ranging from plant-based milk to gluten-free bread fortification, emulsifiers, meat analogs, and functional food ingredients that potentially offer various health benefits while requiring regulatory compliance to ensure food safety and consumer acceptance [8,9,10], Figure 1.

Hemp offers a balanced amino acid profile with relatively low lysine (approx. 3.5 g 100 g^−1^ protein) and tryptophan (approx. 1.0 g 100 g^−1^ protein), low allergenicity, and minimal environmental impact compared with other crops [11]. Despite challenges in mimicking meat’s sensory experience, ongoing research aims to enhance hemp protein’s techno-functional properties through various modification techniques [1,11]. Hemp protein’s versatility extends to applications in plant milk, emulsifiers, gluten-free bread fortification, and plant-based meat production [2]. Additionally, hemp seeds provide essential nutrients, fiber, and bioactive compounds that may offer health benefits and reduce the risk of chronic diseases [8]. As the industrial hemp market grows, further research is needed to optimize its potential in food applications and address remaining challenges [12,13,14]. However, given the growing interest in sustainable and nutritious protein sources, this review aims to comprehensively examine the current state of hemp protein research, its techno-functional properties, and its applications in food systems. This review summarizes recent advances in hemp protein extraction, modification, and characterization; compares nutritional and functional properties with other plant-based proteins; highlights potential health benefits; and discusses current limitations while suggesting directions for future research.

Recent research highlights significant advances in hemp protein extraction, modification, and characterization. Hemp seeds contain 25–30% protein, primarily edestin and albumin, with protein content increasing up to 50% in hempseed cake after oil extraction [15]. Various modification techniques have enhanced hemp protein functionality, including ultrasound, high-pressure homogenization, conjugation, complexation, fibrillation, and enzymatic methods [11]. These modifications improve techno-functional properties such as emulsification, foaming, and gel formation. Hemp proteins also exhibit bioactive properties, with enzymatic hydrolysis producing peptides with antioxidant and antihypertensive effects [15]. The growing interest in hemp protein is driven by its nutritional value, digestibility, low allergenicity, and potential as a sustainable alternative to traditional protein sources [16]. Ongoing research focuses on optimizing extraction processes and exploring novel applications in the food and nutraceutical industries [17].

Hemp protein is a complete, high-fiber, nutrient-rich plant protein with unique functional and health-promoting properties. However, it has a slightly lower protein concentration and lysine content compared with soy and pea proteins. Its low allergenicity and sustainability advantages make it a promising alternative in plant-based nutrition and food applications (Table 1).

Hemp protein contains all essential amino acids with leucine, valine, and phenylalanine being among the most abundant. The protein is highly digestible with in vitro protein digestibility exceeding 88%. Hemp protein is rich in edestin, a storage protein, and exhibits antioxidant activity [23]. Its consumption may contribute to a reduced risk of chronic diseases and support overall health [8]. It contains bioactive peptides with potent antioxidant and antihypertensive effects that reduce oxidative stress and lower blood pressure, benefiting cardiovascular health [24]. Hemp also provides essential omega-3 and omega-6 fatty acids, dietary fiber, and minerals like iron and magnesium, which promote heart, brain, digestive, and immune functions [25]. Additionally, hemp peptides exhibit anti-inflammatory and anticancer properties by modulating key cellular pathways, making hemp protein a promising ingredient for functional foods aimed at preventing chronic diseases and malnutrition [26]. Overall, hemp protein is a sustainable, nutrient-dense source with multifaceted health benefits, and ongoing research continues to explore its full potential in disease prevention and health promotion.

Despite hemp-based meat analogs holding promise as sustainable, nutritious alternatives to animal products, they face several challenges limiting their adoption. Nutritional issues include limited bioavailability of some amino acids and anti-nutritional factors that affect protein quality and digestibility [8]. Sensory drawbacks like hemp’s earthy flavor, color, and texture complicate efforts to replicate meat’s sensory profile, requiring advanced processing techniques [27]. Hence, hemp is emerging as a promising sustainable crop with diverse food, nutraceuticals, and industry applications. However, production hurdles like high raw material costs, inconsistent protein quality, and the need for energy-efficient, scalable methods further restrict commercialization [28]. Also, low consumer awareness, taste concerns, allergenicity, and unclear regulatory frameworks impede market growth [25].

To overcome these barriers, research must focus on improving hemp protein’s nutritional quality through enzymatic treatments, fermentation, and breeding. Processing innovations—such as protein hydrolysis, high-intensity ultrasound, and novel extrusion—are needed to enhance sensory and functional properties. Developing sustainable, cost-effective production and robust supply chains is vital for commercial success. Additionally, consumer education, clear regulatory guidelines, and thorough safety assessments will build trust and acceptance. This review critically examines these challenges and advances, providing a roadmap for future research to fully realize hemp-based meat analogs’ potential in sustainable food systems. Also, it would analyze the contribution of hemp-based analogs to protein, dietary fiber intake, the profile of unsaturated fatty acids, and the presence of essential minerals and bioactive compounds, highlighting their role in promoting human health.

## 2. Methodology

The methodology for this review was designed to ensure a comprehensive and critical synthesis of contemporary research on hemp-based meat analogs. A thorough literature search was conducted in major academic databases, including PubMed, Scopus, Web of Science, and ScienceDirect, covering publications from 2020 to 2025. Search terms combined keywords such as “hemp protein,” “meat analogs,” “plant-based protein,” “nutritional profile,” “techno-functional properties,” “protein blends,” and “health benefits”, using Boolean operators to optimize coverage. Only peer-reviewed original articles, systematic reviews, and authoritative reports in English that directly addressed the technological, sensory, nutritional, or functional aspects of hemp-based meat analogs or blends with other plant proteins were included, with exclusion criteria eliminating non-peer-reviewed literature, non-English texts, and works unrelated to food or nutritional applications of hemp. Selected studies were critically appraised for relevance and methodological quality, and data were systematically extracted regarding study aims, methods, sample characteristics, and significant findings. The extracted data were synthesized thematically to provide an integrated narrative summary, highlighting recent advances in nutritional and techno-functional properties, comparison with other plant-based proteins, health implications, and challenges in product development and market adoption. This transparent methodological approach ensures that the review provides an informative, rigorous, and up-to-date assessment of the role and potential of hemp-based meat analogs in sustainable food systems.

## 3. Comparative Analysis of Hemp Protein Extraction Technologies

The comparative metrics in Table 2 highlight clear performance trade-offs among hemp protein extraction methods. Alkaline extraction–isoelectric precipitation (AE-IP) remains the industrial benchmark, delivering moderate yields (45–60%) and purity (75–85%) at a low unit cost (US $2.50–3.20 kg^−1^ protein) but suffering from reduced functional quality (score 6.5/10) and low solubility (45–60%) due to protein unfolding and disulfide-mediated aggregation under high pH (>10) [9,29]. Consumer acceptance is likewise modest (45–50%) for AE-IP products owing to the resulting gritty texture and limited bioavailability.

Enzymatic extraction offers enhanced performance, achieving yields of 55–70% and a purity of 80–90%, with functional scores of 8.5/10 and a solubility of 75–85% through controlled proteolysis that preserves native structure and generates bioactive peptides (hydrolysis degree increase of 15–25%) [30,31]. However, enzyme costs ($2–5 kg^−1^ material) and longer processing times (4–8 h) raise production costs to US$ per 3.80–4.50 kg^−1^ protein, though consumer acceptance rises to 65–70% due to improved digestibility and mouthfeel [9].

Ultrasound-assisted extraction (UAE) achieves high yields (60–75%) and purity (85–92%) at a moderate cost (US $3.20–4.00 kg^−1^ protein), leveraging acoustic cavitation (up to 1000 atm) to disrupt cell walls with minimal denaturation, resulting in functional scores of 8.0/10 and a solubility of 75–85% [32]. Processing times are short (0.5–1.5 h) with moderate energy consumption (0.25–0.40 kWh/kg).

Microwave-assisted extraction (MAE) combines rapid heating with selective excitation of water molecules, yielding 58–68% of protein with a purity of 80–88%, a functional score of 7.5/10, and a solubility of 70–80%. Energy efficiency is 40–60% higher than conventional methods, and extraction completes in under 0.5 h [33].

Supercritical fluid extraction (SFE) produces the highest purity (90–95%) and functional score (9.0/10) by preserving protein bioactivity, but capital costs are prohibitive (US $300 000–800 000 MT^−1^) and unit costs reach US $5.50–7.20 kg^−1^ protein, limiting its use to niche, high-value applications [34].

Environmental assessments indicate that enzymatic, UAE, and MAE methods incur low water (200–400 L/kg) and carbon footprints (1.2–1.8 kg CO_2_ eq/kg). In contrast, AE-IP has moderate impacts, and SFE achieves very low impacts only at high cost [9]. Overall, enzymatic and ultrasound-assisted extractions emerge as balanced strategies for high-functionality hemp proteins, AE-IP remains most cost-effective for bulk applications, and SFE suits specialty markets.

Interestingly, health-conscious consumers (78%) favor function over price, accepting 15–25% of premiums on hemp versus soy if their functionality matches. The US$847 million market (2023) is growing at 16.8% CAGR to 2030. Profitability arises above 500 t yr^−1^, especially with integrated oil operations (20–30% cost savings). Hemp protein’s carbon footprint (1.2–1.8 kg CO_2_ eq kg^−1^) and water use (200–400 L kg^−1^) are 40–50% lower than whey, appealing to sustainability priorities [35,36].

## 4. Nutritional Profile and Health Benefits

**Figure 2:**
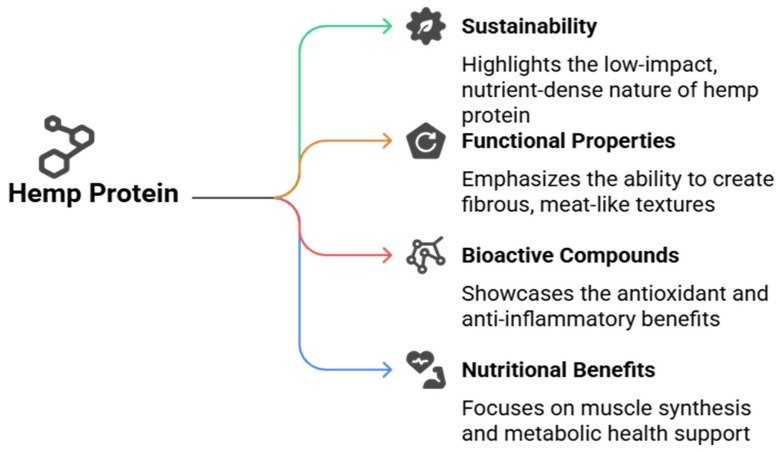
Hemp Protein: A Nutritious and Functional Ingredient for Plant-Based Meat Analogs.

Hemp protein is increasingly recognized as a promising ingredient for plant-based meat analogs due to its favorable nutritional profile, digestibility, and bioactive compounds (Figure 2). Typically, hemp seeds contain 20–25% of protein, with a balanced amino acid composition that includes all nine essential amino acids (limiting amino acids are lysine and tryptophan), making it a rich protein source [12,19]. The primary storage proteins in hemp are edestin and albumin, which are highly digestible proteins contributing to efficient nutrient absorption and utilization in humans [12,37]. This digestibility is crucial for meat analogs aiming to provide a valuable protein comparable to animal sources.

Regarding amino acid composition, hemp protein is rich in arginine, glutamic acid, and branched-chain amino acids, supporting muscle synthesis and metabolic functions. Although its lysine content is somewhat lower than in soy, hemp’s overall amino acid profile remains well balanced for human nutrition [19]. Furthermore, hemp protein contains bioactive peptides released during hydrolysis that exhibit antioxidant, antihypertensive, and anti-inflammatory activities, which may confer additional health benefits beyond basic nutrition [12,37].

Hemp seeds also contribute beneficial lipids, primarily unsaturated fatty acids (25–35%), including omega-3 and omega-6 fatty acids, which support cardiovascular and brain health [12,37]. The natural color and flavor of hemp protein can help regulate the appearance of meat analogs without extensive flavor masking, an advantage over other plant proteins that often require additives to neutralize beany or earthy notes. The brown hue of hemp protein preparations largely reflects the inclusion of hull-derived pigments; protein isolates from dehulled seeds are substantially lighter, underscoring the influence of seed processing on color [27,38]. Additionally, hemp protein exhibits good emulsifying and foaming capacities. However, its gelling ability is lower than that of soy or pea proteins, which may affect the texture of meat analogs and require further optimization [12].

From a health perspective, hemp-based meat analogs provide protein and bioactive compounds with potential therapeutic effects [15]. Antioxidant peptides derived from hemp protein hydrolysates can mitigate oxidative stress, contributing to chronic diseases such as cardiovascular disease, diabetes, and cancer [8]. Moreover, hemp peptides have demonstrated antihypertensive effects by inhibiting angiotensin-converting enzyme (ACE) activity, supporting blood pressure regulation [24]. The presence of dietary fiber, vitamins, and minerals like iron, magnesium, and potassium further enhances the nutritional value of hemp-based products, contributing to digestive health and metabolic balance [26].

The functional properties of hemp protein are vital for its application in meat analogs. However, HPC absorbs less water and requires higher denaturation temperatures than soy protein isolate, influencing processing parameters [15,37]. Combining hemp protein with other plant proteins, such as wheat gluten or chickpea protein, improves texturization and sensory qualities, with formulations like 50:50 HPC–wheat gluten showing superior hardness and consumer acceptability [11,39].

Conclusively, hemp protein offers a nutritionally complete, digestible, and functionally versatile ingredient for plant-based meat analogs. Its bioactive peptides and favorable techno-functional properties support the development of meat substitutes that are both health-promoting and sensory appealing. While challenges remain in optimizing ITS texture and flavor, ongoing research and innovative processing strategies continue to advance hemp’s role as a sustainable and nutritious alternative protein source in the growing market for plant-based meats.

## 5. Textural and Sensory Properties with Protein Blends

Instrumental texture measurements, such as Warner–Bratzler Shear Force (WBSF) and tensile strength, strongly correlate with sensory attributes like chewiness and hardness in plant-based meat analogs. Studies have demonstrated that hemp-based protein blends, particularly when combined with wheat gluten or chickpea protein, can achieve textural profiles closely resembling those of chicken, pork, and beef benchmarks. For example, high-moisture meat analogs (HMMA) containing up to 60% hemp protein concentrate exhibit desirable hardness, resilience, and cutting strength, comparable to animal meat, as both instrumental and sensory analyses confirm. Such formulations provide fibrous structures with a mouthfeel and chewiness that satisfy consumer expectations, highlighting hemp protein’s potential as a functional ingredient for realistic meat analogs [14,37,40]. The optimization of extrusion parameters and protein blend ratios further enhances these textural properties, enabling hemp-based products to mimic meat texture effectively while maintaining good sensory acceptance [40]. The strategies for incorporating hemp protein in plant-based meat alternatives concerning the effects on technological performance, nutritional quality, texture, rheology, and sensory attributes are illustrated in Table 3.

This extensive list demonstrates that blending hemp protein with various plant proteins and fibers significantly improves plant-based meat analogs’ technological, nutritional, mechanical, and sensory properties. These blends enable the development of products with fibrous textures, and consumer-acceptable flavors and mouthfeel, supporting hemp’s role as a versatile, sustainable protein source in the growing plant-based meat market.

In summary, while hemp protein alone provides a robust nutritional and functional foundation, its integration with complementary plant proteins and the application of advanced structuring methods consistently yield superior meat-like textures and consumer acceptance. Blends such as 50:50 hemp–wheat gluten and hemp–chickpea have demonstrated enhanced hardness, chewiness, and fibrous network formation under high-moisture extrusion, outperforming single-source formulations. Moreover, enzymatic cross-linking via transglutaminase further improves network strength and juiciness, particularly in ternary hemp–pea–soy systems. These findings underscore that the most promising HBMAs employ synergistic protein combinations alongside precision processing, guiding future research toward optimizing blend ratios, extrusion parameters, and enzyme treatments to achieve scalable, sensorially convincing, and nutritionally balanced hemp-based meat analogs [51].

Novel technologies for flavor enhancement in plant-based meat products increasingly leverage precision fermentation and targeted enzymatic bioprocessing to produce authentic meat-like tastes. Precision fermentation using engineered microbial strains can biosynthesize heme proteins, savory peptides, and complex aroma volatiles that replicate the depth and umami of animal-derived flavors while masking undesirable off-notes inherent to plant proteins. Concurrently, enzymatic treatments employing lipases and proteases can generate flavor precursors through controlled lipid hydrolysis and protein breakdown, yielding desirable Maillard reaction substrates during cooking. Additionally, advanced encapsulation methods—such as microfluidic-based microencapsulation—stabilize and protect sensitive flavor compounds, enabling their controlled release under specific thermal or pH conditions to mimic the succulence and burst of juiciness found in conventional meats. These integrated biotechnological and formulation strategies offer a pathway to producing plant-based meats with enhanced sensory authenticity and consumer appeal [52].

## 6. Techno-Functional Properties

The techno-functional properties of hemp protein position it as a promising candidate for plant-based meat analogs, owing to its solubility, emulsification, gelation capabilities, and modifiability. This review synthesizes the current understanding of these properties and explores modification strategies that enhance hemp protein’s utility in mimicking meat’s texture and sensory attributes. Protein solubility is a fundamental property affecting the functionality of plant proteins in meat analogs, influencing texture, water retention, and binding capacity. Hemp protein exhibits moderate solubility, which can be limited by its inherent structure and the presence of hydrophobic amino acids. Solubility is pH-dependent, typically increasing at alkaline pH, facilitating better dispersion in food matrices. However, native hemp protein often requires processing interventions to improve solubility for meat analog applications, as poor solubility can impair emulsification and gelation performance [53,54].

Hemp protein boasts a high content of essential amino acids and bioactive peptides that provide health-promoting effects, making it a nutritionally valuable option. Additionally, hemp farming has a significantly lower environmental impact than animal-based protein production, supporting sustainability objectives in formulating plant-based meat analogs [55,56]. The techno-functional characteristics of hemp protein—including moderate solubility, efficient emulsification, and promising gelation ability—supplemented by targeted modification techniques, render it a suitable candidate for use in plant-based meat alternatives. Progress in enzymatic, physical, and fermentation-based modification methods can help address its natural limitations, allowing hemp protein to play a key role in creating sustainable, nutritious, and sensorially satisfying meat alternatives.

### 6.1. Protein Structure and Functionality

Hempseed varieties contain 21.6–28.9% of protein including all essential amino acids [57]. Hemp protein isolates contain 55–76% of protein with a balanced amino acid profile that is relatively low in lysine and tryptophan, while abundant in arginine and sulfur-containing amino acids, including all essential amino acids. The protein is rich in arginine but limited in lysine and tryptophan. Hemp seeds also provide valuable fatty acids, particularly linoleic and α-linolenic acids, and minerals like potassium and iron [57,58]. Hemp protein demonstrates good functional properties, including water and oil holding capacities, foaming, and emulsification, which can be further enhanced through various modification techniques [11]. These characteristics make hemp protein suitable for diverse food applications, such as beverages and bakery products [8,58]. The growing interest in hemp as a sustainable, plant-based protein source is driven by its nutritional value, low allergenicity, and potential health benefits [8,11].

Hydrocolloids, particularly hydroxypropyl methylcellulose (HPMC), play a crucial role in improving the sensory properties of meat and egg analogs. HPMC enhances water retention and oil adsorption, improving the mouthfeel and texture [59]. Other hydrocolloids like xanthan gum and gellan gum also show potential in enhancing textural attributes of meat analogs [60]. The water holding capacity (WHC) is essential for juiciness in meat analogs and can be controlled through marinade composition, with pH and ionic strength influencing water uptake [61]. Non-animal-based liquid additives affect meat analogs’ physicochemical and structural properties, with oil treatment increasing viscoelasticity and WHC, while water treatment decreases these properties [62]. These findings demonstrate the importance of carefully selecting additives and processing conditions to optimize the sensory characteristics of plant-based meat and egg alternatives.

Hemp seed proteins, particularly edestin (11S globulin), exhibit high emulsifying activity and stability but lower gelling capacity compared with soy or pea proteins [63]. Germination and peeling of hemp seeds, combined with defatting, can enhance protein recovery and emulsion properties [64]. In pea proteins, albumin-rich fractions show superior foaming properties, while globulin-rich fractions demonstrate better emulsion stability [65]. The functional performance of plant proteins is influenced by factors such as cultivar, extraction method, and protein content. Industrially produced plant proteins often have a lower solubility and functionality compared with laboratory-produced ones due to protein denaturation during processing [66]. To optimize plant protein functionality for food applications, targeted fractionation techniques can be employed to control protein composition and, consequently, their functional properties [65].

High-moisture extrusion (HME) of plant proteins is an effective method for producing meat-like fibrous structures in plant-based alternatives. The formation of anisotropic structures during HME is attributed to the development of a multiphase system rather than protein alignment at the molecular level [67]. Transglutaminase (TGase) modifications can enhance protein cross-linking and fiber formation, with effects varying depending on protein source and content. The transformation of protein secondary structures, particularly from an α-helix to β-sheet, is crucial for fiber formation [68]. Interactions among proteins, starches, and lipids play a significant role in texturization, with amylopectin and stearic acid synergistically contributing to improved fibrous structures through an “anchor orientation and flexible cross-linking” mechanism [69]. These findings provide insights into the complex processes involved in HME and offer potential strategies for improving the texture and structure of plant-based meat analogs.

Furthermore, combining soy and whey proteins during high-moisture extrusion has improved the texture and morphology of meat analog products [70]. Adding fibers such as pectin or cellulose to plant protein blends like pea and soy can also increase texture quality and water retention, contributing to the fibrous structure needed for meat-like products [71]. Additionally, optimizing the high-moisture extrusion process makes it possible to create fibrous meat analogs from a range of plant proteins, including hemp [72]. Collectively, these studies indicate that blending plant proteins leads to a wider variety of textures and functions in meat analogs, thus providing greater potential for innovation and customization in product development.

Emulsifying properties are critical for stabilizing fat and water phases in plant-based meat products, contributing to juiciness and mouthfeel. Hemp protein has demonstrated good emulsifying activity due to its amphiphilic nature, enabling it to adsorb at oil–water interfaces and stabilize emulsions. The balance of hydrophilic and hydrophobic residues in hemp protein supports this function, although emulsification efficiency can be enhanced by physical or enzymatic treatments that expose more reactive groups [54,73]. Improved emulsification contributes to the characteristic juiciness and fat distribution in meat analogs.

Gelation—the ability to form three-dimensional protein networks—is essential for replicating meat’s fibrous, chewy texture. Hemp protein forms gels upon heating or enzymatic cross-linking. Still, the gel’s strength and elasticity may be lower than those of soy or wheat gluten proteins commonly used in meat analogs. Modification strategies such as enzymatic hydrolysis, high-pressure processing, or blending with other proteins or polysaccharides can improve gelation properties, enabling better texture mimicry [53,73]. The gelation behavior is influenced by protein concentration, pH, ionic strength, and processing conditions.

### 6.2. Impact of Processing

Extrusion conditions play a crucial role in determining the characteristics of hemp-based meat analogs. Increasing the feed moisture content tends to reduce the hardness and chewiness of the final extrudates [37,50,74]. In contrast, elevated processing temperatures contribute to stronger fiber development and improved structural formation [50,75]. Higher temperatures also promote greater protein denaturation, which enhances fiber strength and overall texturization. Specifically, the combination of high moisture levels (65%), elevated barrel temperatures (170 °C), and low screw speeds (150 rpm) leads to low specific mechanical energy input alongside a high degree of texturization [74]. Moreover, increased processing temperatures decrease the angle between fibrous structures and the extrusion flow direction, fostering more organized secondary structures and greater exposure of sulfhydryl (SH) groups and tryptophan residues [75]. These observations underline the capability of hemp protein to serve as an adequate substitute for soy in producing high-moisture meat analogs [76]. A recent review further elaborates on the advancement of plant-based meat analogs by examining the impact of ingredients, structuring techniques, and processing parameters on replicating meat-like texture, while also highlighting emerging technologies such as extrusion and 3D printing aimed at enhancing sensory qualities and nutritional value to improve consumer acceptance and sustainability [77].

The dry fractionation of plant proteins offers advantages over wet extraction methods for producing meat analogs. Dry-fractionated proteins exhibit higher solubility, foaming ability, and gelling properties. When used in meat analogs, dry-fractionated pea protein resulted in products with lower hardness but higher oil absorption capacity compared with isolates [78]. For hemp proteins, alkaline extraction–isoelectric precipitation (AE-IEP) and salt extraction (SE) methods both yield high-purity isolates, but with differing structural and functional properties [9,79]. SE-extracted hemp protein isolate showed higher solubility and thermal stability than AE-IEP extracted isolate [79]. However, hemp protein isolates generally demonstrate poor solubility at neutral pH compared with soy and pea isolates, though they exhibit comparable gel strength to soy protein [9]. These findings highlight the importance of the extraction method in determining protein functionality for food applications.

Interestingly, several modification approaches have been established to optimize hemp protein for meat analog production. Enzymatic hydrolysis can improve the functional properties of plant proteins, including hemp protein, by modifying their structure and generating peptides with enhanced solubility, emulsifying capacity, and antioxidant activity [80,81]. The degree of hydrolysis, pH, and enzyme type are crucial factors affecting these improvements [81]. Hemp seed protein contains various fractions (2S, 7S, and 11S) with different functional properties, with the 2S fraction showing superior solubility and emulsifying activity [63]. Enzymatic hydrolysis of hemp protein improves its antioxidant properties by generating peptides with exposed functional groups [82].

Additionally, a novel high-pressure homogenization-assisted pH-shift strategy can further enhance hemp protein solubility and interfacial absorption by increasing structural flexibility, surface hydrophobicity, and reducing particle size [83]. Similarly, the enzymatic hydrolysis of lupin proteins improves their solubility and emulsifying properties [84]. These modifications in protein structure and functionality can broaden the potential applications of hemp protein in food systems.

High-intensity ultrasound treatment can improve the functional properties of hemp seed protein isolate by modifying its structure and increasing surface hydrophobicity [85], improving its solubility, emulsifying properties, and functional properties [32,86]. Ultrasound treatment and complexation with chlorogenic acid can modify hemp seed protein’s structural and functional properties [87]. Also, ultrasound and pH shifting treatments can improve the functional properties of hemp protein concentrate by unfolding protein structures and increasing surface hydrophobicity and reactive sites [88]. Manothermosonication, high-pressure homogenization, and pH-shifting can improve the techno-functionality and digestibility of hemp protein [89]. High-pressure homogenization combined with pH shift treatment improves hempseed protein’s solubility and interfacial absorption [83]. High-pressure processing and ultrasound-assisted extraction can improve protein recovery and purity from industrial hemp waste [90].

Recent research has investigated the blending of different plant proteins to improve the functional qualities of meat analogs. For example, when pea and lentil proteins are mixed, they demonstrate a linear mixing effect with both transglutaminase- and heat-induced gels, whereas blends containing hemp display a non-linear behavior [48]. In pea–wheat protein composites, increasing the proportion of pea protein has been shown to boost hardness and chewiness in meat analog products [91]. Incorporating dietary fibers with plant proteins such as soy and pea can further enhance texture, water retention, and nutritional value in plant-based meat-like products [92]. Hemp protein concentrate is capable of replacing up to 60% of soy protein isolate in high-moisture meat analogs (HMMAs), which influences both water absorption and textural properties [37]. Using blends of soy and pea protein isolates in HMMAs provides better visual and textural attributes than pea-only counterparts, with pea protein serving as a possible tenderizer in soy-based formulations [93].

Solid-state fermentation (SSF) has emerged as a promising approach to enhance plant-based proteins, particularly for meat analogs. SSF improves protein digestibility, reduces anti-nutritional factors, and enhances flavor compounds [94,95]. Fermentation can improve digestibility, reduce anti-nutritional factors, and enhance the flavor and texture of plant-based proteins like hemp, making them more suitable for meat analogs [96]. This biotechnological process offers advantages over submerged fermentation in energy and water efficiency [94]. For hemp protein specifically, SSF using Lactobacillus plantarum and Bacillus subtilis has increased protein recovery, improved solubility, and enhanced functional properties [97]. SSF also augments protein content through microbial hydrolysis, refines amino acid profiles, and generates bioactive peptides [98]. Moreover, fermentation with microorganisms like B. subtilis and L. plantarum can reduce allergenicity and improve the safety aspects of plant-based meat alternatives [99]. These findings highlight SSF’s potential to address key limitations in current meat analogs and advance sustainable protein systems.

## 7. Applications in Food Products

Hemp is increasingly recognized as a sustainable and versatile crop with valuable applications in the food industry due to its seeds’ high protein, polyunsaturated fatty acid, and fiber content [100]. Hemp protein is utilized in plant-based milks, meat alternatives, gluten-free bread, and as an emulsifier [2]. Its antioxidants, including polyphenols, may reduce anxiety and lower chronic disease risk [100]. While hemp-based foods are promoted for health benefits, consumer acceptance varies [101]. The global hemp market includes over 25,000 products spanning food, beverages, and nutraceuticals [102], though regulatory challenges related to THC content continue to affect product safety [101].

### 7.1. Nutritional and Health Implications

Table 4 highlights hemp’s rich nutritional profile, health benefits, potential as a sustainable, functional ingredient, consumer acceptance, and regulatory compliance in meat analogs.

Hemp protein is a sustainable, nutritious alternative to animal proteins, offering essential nutrients, fiber, and antioxidants [8]. While plant-based replacements can boost iron and folate intake owing to their high content of non-heme iron, which might be beneficial with co-consumption of vitamin C, they may reduce vitamin B_12_ and iodine levels [109]. Hemp’s nutritional value, similar to soy, supports its use in plant-based milk, meat alternatives, and gluten-free products [2]. However, when substituting animal proteins, it is crucial to ensure adequate micronutrient intake, as some meat alternatives may lack the full benefits of traditional plant-based diets [110]. Overall, hemp’s sustainability, versatility, and nutrition make it a promising ingredient for healthier, eco-friendly foods [10,111]. Table 5 summarizes the micronutrient enrichment from replacing meat with hemp protein, highlighting the increase in mineral content and nutritional value that supports health-conscious and flexitarian diets.

### 7.2. Technological Innovations

Enzyme-Assisted Structuring: TGase and other enzymes enable superior textural and structural outcomes, facilitating the creation of highly fibrous, meat-like analogs from diverse protein blends [19]. Regional Adaptation: Hemp’s adaptability to various climates enables regional production, reducing reliance on soy and supporting local food systems [37]. Sustainable Processing: Advances in dry and wet fractionation, high-moisture extrusion, and protein blending drive the development of minimally processed, clean-label HBMAs [14]. Table 6 highlights how technological, agricultural, and sustainable processing advancements integrate hemp into food product innovation.

Interestingly, innovations in HBMAs are rapidly expanding beyond traditional extrusion and enzyme-assisted methods to incorporate cutting-edge biotechnologies, advanced processing techniques, and sustainable practices. By integrating precision fermentation, 3D food printing, bioactive fortification, and novel processing technologies, researchers can enhance flavor, texture, and nutritional value while minimizing environmental impact. The following approaches outline these next-generation strategies for developing HBMAs that meet consumer demands for authenticity, functionality, and clean-label credentials. These innovations could be summarized by the following:

• Harnessing precision fermentation via specific microbial strains to develop natural flavor compounds—such as heme analogs, savory peptides, and aroma volatiles—can impart authentic meaty notes and mask any lingering off-notes typical of plant proteins. This approach allows the creation of “fermented hemp bases” that deliver elevated umami and complexity, enhancing consumer acceptance [95]. Also, the use of 3D printing technology enables the creation of customizable fibrous structures and marbling patterns that mimic muscle tissue and fat in animal meat. By controlling deposition and layering of hemp protein-based pastes, it is possible to reproduce the heterogeneity of real meat, catering to various culinary traditions and consumer preferences [37].
• Regrading fortification with functional bioactives, incorporating functional ingredients such as plant-derived omega-3 fatty acids, antioxidants (e.g., polyphenols, tocopherols), dietary fibers (e.g., beta-glucans), and natural iron salts can boost the nutritional profile of HBMAs, aligning with trends for health and functional foods [115]. Additionally, applying pulsed electric field (PEF) or ultrasound during hemp protein processing can modify protein structure, enhance hydration, improve emulsifying properties, and promote better integration of fats and flavors. This leads to superior textural outcomes, reduced ingredient usage, and an enhanced mouthfeel [52].
• On the other hand, innovative use of by-products such as hemp hulls, press cake, or microgreens as ingredients in meat analogs can close resource loops, enhance dietary fiber content, and add unique flavors. Integrating these fractions supports sustainability and zero-waste initiatives in food production [116]. Moreover, encapsulating natural colorants, flavors, or micronutrients using techniques like microencapsulation or liposomal delivery can provide targeted release during cooking or eating, helping to stabilize sensitive components and further mimic the sensory experience of animal meats [117].

### 7.3. Market and Consumer Trends

• Product Diversification: The market is shifting from soy-dominated analogs to diversified products incorporating hemp, pea, chickpea, and other regional proteins [20].
• Environmental Sustainability: Hemp cultivation requires less water, pesticides, and land compared with many other crops, making it a sustainable ingredient for future food systems [13].

## 8. Challenges and Future Directions

### 8.1. Product Diversification

The plant-based meat analog market is transforming from soy-centric formulations to a more diverse range of protein sources, including hemp, pea, chickpea, and various regional legumes [118]. Soy protein, long favored for its functional properties and advantageous amino acid profile, faces challenges related to allergenicity, environmental concerns associated with soy monoculture, and consumer demands for diversified and novel ingredients [119]. Consequently, alternative proteins such as pea and chickpea have gained traction due to their favorable sensory and functional profiles. In contrast, hemp protein is prized for its exceptional nutritional composition, including essential fatty acids, fiber, and bioactive peptides [120].

The diversification trend is shaped by the increasing adoption of flexitarian and plant-forward diets, motivating demand for meat analogs with enhanced nutritional and sensory attributes as alternatives to traditional meat [121]. Regional proteins offer further benefits by enabling localized supply chains and fostering food sovereignty, which appeals to consumers emphasizing sustainability and provenance [122]. Importantly, blending different plant proteins—such as combining hemp with pea or chickpea proteins—has been shown to improve textural properties like gelation and emulsification, resulting in meat analogs with improved fibrousness and juiciness that more closely mimic conventional meat [123].

Such multi-protein composite strategies overcome the limitations inherent in individual protein sources and enable product customization to target specific dietary needs and sensory preferences [121]. Therefore, product diversification represents a key innovation driver in the plant-based meat sector, expanding functional possibilities and market reach [124].

### 8.2. Environmental Sustainability

Environmental sustainability is key in the plant-based food sector’s ingredient choice and consumer preferences. Hemp cultivation uses significantly less water, fertilizer, and land than soy and animal proteins, making it an eco-friendly crop [35]. Its high biomass yield and ability to grow on marginal soils promote efficient land use and reduce deforestation and biodiversity loss [125]. Additionally, hemp’s deep roots improve soil health, supporting regenerative agriculture [126]. These traits align with consumer demand for low-impact, minimally processed foods, while hemp by-products aid circular economy efforts by reducing waste and enabling multi-industry uses [127].

### 8.3. Future Prospects

Despite its benefits, hemp-based meat analogs face challenges in consumer acceptance due to earthy and unfamiliar flavors, requiring improved processing and flavor masking [20,44]. Regulatory variability, especially regarding THC limits and cultivation, also restricts market access in some regions [128]. To overcome these barriers, technologies like enzyme-assisted structuring, fermentation, and high-moisture extrusion are used to enhance texture and flavor [19,77]. Additionally, consumer education on hemp’s nutritional and environmental advantages is vital for market growth [22,55]. The shift toward diverse plant proteins like hemp, driven by sustainability and health concerns, highlights hemp’s potential role in future meat analogs, provided sensory and regulatory challenges are addressed through innovation and transparent communication [27,121].

Building on these strategies, next-generation innovations are rapidly transforming hemp protein applications. Advances such as AI-optimized recipe development, 3D and bioprinting technologies for precise texture customization, and enhanced solid-state fermentation with targeted microbial strains are improving both sensory qualities and digestibility of hemp-based products. Additionally, biotransformation techniques like germination combined with high-intensity ultrasound are generating nanoaggregates with superior functional properties. At the same time, precision fermentation facilitates the production of hemp-derived bioactive compounds mimicking key nutritional factors. Blockchain-enabled supply chain transparency and hybrid protein blends that combine hemp with other plant proteins further optimize product appeal and regulatory compliance. Enhanced flavor masking through natural compounds and fermentation is mitigating hemp’s characteristic earthy taste, supporting broader consumer acceptance. These technological and educational advances, coupled with targeted marketing to health- and eco-conscious demographics, are projected to accelerate hemp protein’s market value toward 2.6 B$ by 2033, integrating hemp more fully into mainstream food categories such as beverages, bars, dairy alternatives, and hybrid meat products. Ultimately, the success of hemp-based meat analogs hinges on continued innovation, regulatory harmonization, and clear consumer communication to realize their sustainable and nutritional potential on a global scale.

## 9. Conclusions

Hemp-based meat analogs (HBMAs) have emerged as an up-and-coming category within the plant-based protein sector, offering a compelling combination of nutritional, functional, and environmental advantages. This review highlights that hemp protein can produce meat analogs with desirable textural, sensory, and nutritional profiles when used alone or in strategic blends with other plant proteins. Advances in processing technologies—such as high-moisture extrusion and enzyme-assisted structuring—have enabled the creation of products that closely mimic the fibrous texture and mouthfeel of conventional meats. Furthermore, hemp’s favorable amino acid composition, high fiber content, and sustainable cultivation practices position it as a key ingredient for the future of alternative proteins. Despite significant progress, further research is needed to optimize hemp protein extraction and processing to enhance functionality and reduce off-flavors. Exploring synergistic hemp blends with other plant proteins like pea and chickpea can improve sensory and nutritional qualities. Advances in structuring agents, enzymes, and processing will help better replicate meat textures and boost consumer acceptance. Long-term clinical studies must confirm health benefits and address allergenicity or digestibility concerns. Additionally, comprehensive sustainability assessments and navigating regulatory frameworks are crucial for large-scale adoption. Overall, hemp-based meat analogs hold great promise for sustainable, nutritious plant-based foods, and interdisciplinary collaboration is key to unlocking their full potential.

## Figures and Tables

**Table 1:** Summarizing hemp protein’s nutritional and functional attributes versus other common plant-based proteins (soy and pea).

**Attribute**	**Hemp Protein**	**Soy Protein**	**Pea Protein**	**Meat Protein**	**Ref.**
Protein Content	~51% (in hemp protein powder)	61–91% (varies by source)	77–81%	25–30%	[9]
Amino Acid Profile	Complete protein with all 9 essential amino acids but relatively low in lysine; rich in sulfur-containing amino acids and arginine	Complete protein that meets essential amino acid requirements; higher lysine content	Complete protein that meets essential amino acid requirements; higher lysine content	Complete with optimal proportions of all essential amino acids and notably high in lysine, methionine, and leucine.	[16]
PDCAAS Score	Scores 49–53%	Scores 98–100%	Scores 83–91%	100%	[18]
DIAAS Score	Scores 60 ≤ 75	Scores > 75	-	>100	
Fiber Content	High fiber content that aids digestion and gut health	Low fiber (typically isolated protein)	Low fiber (typically isolated protein)	No dietary fiber	[19]
Digestibility	Naturally easy to digest with less kidney stress	Good digestibility but may cause allergies in some cases	Good digestibility; hypoallergenic	Easily digested and absorbed with minimal anti-nutritional factors	[11,16]
Functional Properties	Good emulsification and gelation; unique cysteine-rich composition aids food processing	Widely used for emulsification and gelation; versatile	Good emulsification and gelation; popular in meat analogs	Superior water-holding, emulsification, and gelation used in processed meats	[16,20]
Additional Nutrients	Rich in omega-3 fatty acids, magnesium, iron, zinc, and anti-inflammatory properties	Contains isoflavones and minerals	Contains iron and other minerals	Sources of vitamin B12, heme iron, zinc, creatine, and taurine	[8]
Allergenicity	Low allergenicity; soy and dairy-free	Potential allergen (soy)	Generally low allergenicity	Allergies are rare vs. plant protein allergens	[21]
Environmental Impact	Cultivation stands out for its minimal water and pesticide requirements, soil remediation capabilities, and low environmental footprint, making it an environmentally friendly protein source	While nutritionally valuable, it has sustainability challenges related to pesticide use, GMO crops, deforestation, and processing intensity	It offers sustainability advantages through nitrogen fixation and has a moderate environmental impact, though it generally requires more water and pesticides than hemp	Highest resource use—significant greenhouse gas emissions and land and water use compared with plant proteins	[22]

PDCAAS Score: Protein Digestibility-Corrected Amino Acid Score, DIAAS Score: Digestible Indispensable Amino Acid Score, -: Not available data.

**Table 2:** Comparative Performance Metrics of Hemp Protein Extraction Methods.

**Parameters**	**Extraction Method**				
	**AE-IP**	**EE**	**UAE**	**MAE**	**SFE**
Yield (% *w*/*w*)	45–60	55–70	60–75	58–68	40–55
Purity (%)	75–85	80–90	85–92	80–88	90–95
Processing Time (h)	2–4	4–8	0.5–1.5	0.17–0.5	1–3
Energy Cost ($/kg)	0.15–0.25	0.20–0.30	0.25–0.40	0.18–0.28	0.50–0.80
Equipment Cost ($/MT capacity)	50,000–150,000	80,000–200,000	120,000–250,000	100,000–220,000	300,000–800,000
Total Cost ($/kg protein)	2.50–3.20	3.80–4.50	3.20–4.00	3.00–3.80	5.50–7.20
Solubility (%)	45–60	75–85	75–85	70–80	80–90
Consumer Acceptance (%)	45–50	65–70	60–65	55–60	70–75
Functional Properties Score *	6.5/10	8.5/10	8.0/10	7.5/10	9.0/10
Environmental Impact **	Moderate	Low	Low	Low	Very Low
Refs.	[9,29]	[30,31]	[32]	[33]	[34]

AE-IP: Alkaline Extraction–Isoelectric Precipitation, EE: Enzymatic Extraction, UAE: Ultrasound-Assisted Extraction, MAE: Microwave-Assisted Extraction, SFE: Supercritical Fluid Extraction, * Functional Properties Score based on solubility, emulsification, foaming, and gelation properties, ** Environmental Impact: Combined assessment of carbon footprint, water usage, and energy consumption.

**Table 3:** Protein Blending Strategies Using Hemp Protein in Plant-Based Meat Analogs: Effects on Technological, Nutritional, Mechanical, Rheological, and Sensory Properties.

**Protein Blend and Ratio**	**Technological Changes and Processing Notes**	**Nutritional Impact**	**Mechanical and Rheological Properties**	**Sensory (Appearance and flavor Attributes)**	**Ref.**
Hemp + Wheat Gluten (90:10, 50:50)	Improved texturization, water retention, and extrusion stability	Complements lysine deficiency in hemp; balanced amino acids	Increased hardness, chewiness, resilience, and fibrous texture	Uniform brown color, visible fibers, juiciness, earthy, nutty, slight beany, meaty, some off-notes	[19,39]
Hemp + Chickpea Protein (50:50)	Enhanced emulsification and gelation; soy-free formulation	Improved lysine and sulfur amino acid profile	Good chewiness and firmness; stable gel formation	Light brown-tan color, glossy, retains structure, beany, slightly grassy, mild nuttiness	[39]
Soy + Hemp + Wheat + Transglutaminase (TGase)	TGase cross-linking increases protein network strength and water holding capacity	Improved protein digestibility and quality	Pronounced fibrous structure; improved mouthfeel and juiciness	Browns, develops grill-marks, and has fiber alignment; flavor has enhanced umami, reduced off-notes, and is rich	[19]
Hemp Protein Concentrate (HPC) alone (up to 60%)	Requires a higher denaturation temperature; lower water absorption than soy	Complete protein but lower lysine than soy	Moderate hardness and chewiness; less fibrous than blends	Natural brown color with mild nutty flavor; less gloss, strong earthy, grassy, and mild nutty	[37,39]
Hemp + Soy Protein Isolate (SPI)	Improved gelation and emulsification; better processing stability	Enhanced essential amino acid profile; increased digestibility	Increased elasticity and firmness; improved texture uniformity	Brown shade, surface gloss, umami, mild earthy, soy masking ability	[15,37]
Hemp + Pea Protein (4:1 with maize starch)	Improved solubility, emulsification, and foaming properties	Complementary amino acid profile; increased lysine content	Balanced texture with improved chewiness and cohesiveness	Generally well accepted, mild flavor profile, uniform pale color, smooth surface, mild earthy, subdued beany, less bitterness	[12]
Hemp + Oat Fiber	Produces fibrous structure and brown color; influences texture via moisture	Adds dietary fiber and healthy fats	Fibrous texture; moisture content affects hardness and chewiness	Positive texture; natural color regulation, visible fibers, irregular surface, mild earthy, faint grassy	[41]
Hemp + Faba Bean Protein	Complementary amino acids; improved emulsification	Balanced amino acids; enhanced protein quality	Good gelation and foam stability	Improved sensory profile, matte beige color, marbling simulation, earthy, faint bean, masks hemp flavor	[42,43]
Hemp + Yellow Pea Protein	Synergistic effects on texture and nutrition	Improved lysine and sulfur amino acid content	Enhanced fibrousness and chewiness	High consumer acceptance, good red-brown, surface shine, minimal beany, mild earthy notes, balanced flavor	[44]
Hemp + Buckwheat Protein	Improved emulsification and gelling properties	Enhanced mineral and amino acid profile	Moderate gel strength; improved water holding	Mild flavor, acceptable texture, slightly brown color	[45]
Hemp + Rice Protein	Improved solubility and emulsification	Complementary amino acids; hypoallergenic	Balanced texture; moderate hardness	Mild flavor; good acceptance, mild earthy, subdued beany, less bitterness	[46]
Hemp + Pea + Wheat Gluten (Ternary blend)	Synergistic effects on extrusion and texture	Balanced amino acid profile (abundant in arginine and sulfur-containing amino acids); improved digestibility	Enhanced fibrous texture; improved chewiness	High consumer acceptance, brown color, earthy, nutty, slight beany flavor	[47]
Hemp + pea + Chickpea Protein (Ternary blend)	Improved gelation, emulsification, and texture	Enhanced amino acid balance; soy-free option	Increased hardness and chewiness	Positive sensory evaluation, beany flavor	[48,49]
Hemp + Soy + Pea Protein	Improved protein network formation and texture	Enhanced essential amino acids; increased digestibility	Increased firmness and elasticity	Good sensory acceptance, robust meaty, umami, slightly nutty, and beany	[44]
Hemp + Wheat Gluten + Chickpea Protein	Improved extrusion behavior and texture	Balanced amino acid profile (low in lysine and tryptophan); improved fiber content	Increased hardness, chewiness, and resilience	Browning, grill surface, structural fidelity, balanced, mild earthy, meaty	[39]
Hemp + Pea + Oat Fiber	Improved fiber content and texture	Increased dietary fiber and balanced amino acids	Fibrous texture; improved water retention	Deep, uniform brown color, earthy, mild nutty, light oat undertone, subtle beany notes	[50]
Hemp + Pea + Soy + Wheat Gluten + TGase	Enzymatic cross-linking enhances texture and water holding	Enhanced protein quality and digestibility	Pronounced fibrous structure; improved juiciness	Excellent sensory acceptance, rich, dark brown hue; robust meaty, pronounced umami, slightly nutty and beany; minimal off-notes	[14,19]
Hemp + Pea + Chickpea + Wheat Gluten	Improved extrusion and texturization	Balanced amino acid and fiber content	Enhanced chewiness and texture uniformity	Brown color, faint golden tint from chickpea, balanced earthy, light nutty and chickpea flavor, mild beany tones	[39]

**Table 4:** Nutritional and Health Implications of Hemp-Based Meat Analogs, including challenges in consumer acceptance and regulatory standards.

**Category**	**Details**	**Refs.**
Nutritional Content	- Rich source of essential amino acids, good protein, fiber, and polyunsaturated fatty acids (omega-3 and omega-6). - Contains bioactive compounds such as lignanamides and polyphenols with antioxidant properties.	[103,104,105]
Health Benefits	- Antioxidants and bioactives may reduce anxiety, support cardiovascular health, and lower the risk of chronic diseases. - Dietary fiber contributes to digestive health. - Potential neuroprotective effects via acetylcholinesterase inhibition.	[106,107]
Functional Properties in Meat Analogs	- Moderate solubility, emulsification, and gelation can be improved by technological modification. - Enables production of textured, fibrous plant-based meat analogs with enhanced sensory qualities.	[104,105]
Sustainability	- Low environmental footprint compared with animal protein due to reduced land and water use and greenhouse gas emissions. - Supports zero-waste goals through the valorization of hemp seed meals and its by-products.	[108]
Consumer Acceptance	- Marketed health benefits are increasing in popularity. - Consumer perception varies due to unfamiliarity and sensory preferences. - Some concern over taste, texture, and product novelty limits acceptance.	[105,108]
Regulatory Challenges	- Strict regulations on tetrahydrocannabinol (THC) content restrict cultivation and food use in some regions. - Ensuring compliance with THC limits (commonly <0.3%) is critical for food safety and legal status. - Regulatory uncertainties can hinder product development and market expansion.	[104,105,108]

**Table 5:** Micronutrient Enrichment from Replacing Meat with Hemp Protein.

**Nutrients**	**Meat (e.g., Turkey, Fish)**	**Hemp-Enriched Meat** **Analogs/Hemp Protein**	**Impact on Nutrition and Health Benefits**	**Refs**
Minerals	- Variable mineral levels (Ca, K, Mg, Fe)	- Significant increases in calcium, potassium, magnesium, and iron	- Improves mineral intake essential for bone health, metabolism, and oxygen transport	[112,113]
Vitamins	- Meat provides certain fat-soluble vitamins	- Increased vitamins A, D, E, and B group vitamins	- Contributes to immune function, antioxidant protection, and energy metabolism	[112]
Bioactive Compounds	- Low or absent	- Polyphenols and antioxidants present in hemp	- May reduce inflammation and chronic disease risk	[112]
Consumer Health Implications	- Traditional benefits of meat proteins	- Improved mineral bioavailability and antioxidant intake from hemp	- Potential to reduce anemia risk, improve antioxidant status	[114]
Sustainability and Diet Support	- Animal protein has a higher environmental impact	- Plant-based hemp protein supports sustainable food systems	- Aligns with flexitarian and health-conscious dietary patterns	[112]

**Table 6:** Applications of hemp in food products.

**Application Area**	**Description**	**Benefits**	**Refs.**
Enzyme-Assisted Structuring	- Use of enzymes like transglutaminase (TGase) to create fibrous, meat-like textures from hemp protein blends	- Enhances the texture quality of plant-based meat analogs, producing highly fibrous structures	[19]
Regional Adaptation	- Hemp’s ability to grow in diverse climates enables local cultivation	- Supports localized production, reduces reliance on soy imports, and strengthens regional food systems	[37]
Sustainable Processing	- Techniques such as dry and wet fractionation, high-moisture extrusion, and protein blending	- Enables minimally processed, clean-label hemp-based meat analogs that meet consumer demand for natural and eco-friendly products	[14]

## Data Availability

The original contributions presented in this study are included in the article. Further inquiries can be directed at the corresponding author.
